# Comparative proteomic analysis of extracellular proteins expressed by various clonal types of *Staphylococcus aureus* and during planktonic growth and biofilm development

**DOI:** 10.3389/fmicb.2015.00524

**Published:** 2015-06-03

**Authors:** Salman S. Atshan, Mariana N. Shamsudin, Zamberi Sekawi, Leslie T. Thian Lung, Fatemeh Barantalab, Yun K. Liew, Mateg Ali Alreshidi, Salwa A. Abduljaleel, Rukman A. Hamat

**Affiliations:** ^1^Department of Medical Microbiology and Parasitology, Faculty of Medicine and Health Sciences, Universiti Putra MalaysiaSerdang, Malaysia; ^2^Department of Medical Laboratory Sciences, University College of Humanity StudiesNajaf, Iraq; ^3^Department of Clinical Laboratory Sciences, Faculty of Pharmacy, Basrah UniversityBasrah, Iraq; ^4^Department of Immunology, Faculty of Medicine and Health Science, Universiti Putra MalaysiaSerdang, Malaysia; ^5^Department of Basic Medical Sciences, Faculty of Medicine, Sulaiman Alrajhi CollegesAlbukairiyah, Saudi Arabia; ^6^Department of Biology, Faculty of Science, Basrah UniversityBasrah, Iraq

**Keywords:** biofilm, clone types, *S. aureus*, 2DGE, LC-MS/MS

## Abstract

*Staphylococcus aureus* is well known for its biofilm formation with rapid emergence of new clones circulating worldwide. The main objectives of the study were (1) to identify possible differences in protein expression among various and closely related clonal types of *S. aureus*, (2) to establish the differences in protein expression in terms of size of protein spots and its intensities between bacteria which are grown statically (biofilm formation) with that of under aeration and agitation, and (3) to compare the differences in protein expression as a function of time (in hours). In this study, we selected six clinical isolates comprising two similar (MRSA-527 and MRSA-524) and four different (MRSA-139, MSSA-12E, MSSA-22d, and MSSA-10E) types identified by *spa* typing, MLST and SCCmec typing. We performed 2D gel migration comparison. Also, two MRSA isolates (527 and 139) were selected to determine quantitative changes in the level of extracellular proteins at different biofilm growth time points of 12, 24, and 48 h. The study was done using a strategy that combines 2-DGE and LC-MS/MS analysis for absolute quantification and identification of the extracellular proteins. The 2DGE revealed that the proteomic profiles for the isolates belonging to the similar *spa*, MLST, and SCCmec types were still quite different. Among the extracellular proteins secreted at different time points of biofilm formation, significant changes in protein expression were observed at 48 h incubation as compared to the exponential growth at 12 h incubation. The main conclusion of the work is that the authors do observe differences among isolates, and growth conditions do influence the protein content at different time points of biofilm formation.

## Introduction

*Staphylococcus aureus* is one of the top nosocomial and public health pathogens and causes a wide range of infections, ranging from mild to fatal diseases (Gordon and Lowy, 2008). *S. aureus* clones are highly adaptable and have the ability to form structures known as biofilms, leading to surface colonization and the creation of a niche where the bacteria appear more resistant to host defenses and antimicrobials (Costerton et al., 1999). The process of colonization is initiated by the attachment of *S. aureus* to the surfaces, which is mediated by adhesion factors including “Microbial Surface Components Recognizing Adhesive Matrix Molecules” (MSCRAMMs) (Van Belkum et al., 2009), and secreted expanded-repertoire adhesive molecules (SERAMs) such as the extracellular adhesive protein (Eap), extracellular fibrinogen-binding protein (Efb), and extracellular matrix protein (Emp) (Clarke and Foster, 2006). The cell wall-associated virulence factors and extracellular proteins are controlled by the accessory gene regulator *agr* and the staphylococcal accessory regulator *sar* (Novick, 2000; Gotz, 2002). The *agr* locus regulates the expression of cell wall-associated proteins and secreted exoproteins in response to the density of the bacterial population (Otto, 2001). In addition to two regulatory systems, the alternative sigma factor has been identified as one of the most important virulence regulatory genes, which can regulate the expression of several exoproteins and surface proteins in response to changing environmental conditions. The *sigB* operon of *S. aureus* represents a global regulatory system and has been shown to be intimately involved in biofilm formation and enables the organism to deal with environmental stress (Lauderdale et al., 2009).

Two-dimensional gel electrophoresis (2DGE) is a famous traditional tool that is widely applied for protein separation and quantitation (Tonella et al., 2001; Gupta et al., 2002; Rosen and Ron, 2002; Scherl et al., 2005; Resch et al., 2006). This technique was initially applied on biological fluids to identify potential biomarkers and study model organisms such as *Escherichia coli* and *Bacillus subtilis*. However, to date, studies on the use of proteomic techniques to quantify the protein content of genotypically different clones at multiple time points are still lacking. This manuscript aims at comparing extracellular protein production for six different *S. aureus* isolates. To this extend, we decided to first analyse the differences by using 2D gel migration and followed by liquid chromatography tandem mass spectrometry (LC-MS/MS) to identify proteins that are differentially produced at different time points of biofilm formation. The goal of this study could be of great help in the characterization of genotypically different clones in terms of types of protein involved and could provide potential biomarkers in identifying specific MRSA or MSSA biofilm producers among identical *spa* types in a clinical environment.

## Materials and methods

### Bacterial strains and culture conditions

In this study, six clinical *S. aureus* isolates were subjected to 2D gel sodium dodecyl sulfate-polyacrylamide gel electrophoresis (2DG-SDS-PAGE) for comparative secretomic analysis. These isolates (Table 1) were characterized into different clones through SCCmec, *spa*, and MLST typing. In addition, two representative MRSA isolates were selected (527 and 139) for proteomic analyses at different biofilm growth time points of 12, 24, and 48 h in the second experiment. The liquid chromatography tandem mass spectrometry (LC-MS/MS) was then used to identify proteins which were differentially produced at different times of biofilm formation for the only one representative isolate (MRSA-527).

**Table 1:** **Characteristics of isolates used in this study**.

**Strains no**.	***Spa* types**	**MLST**		**SCCmec**	**Isolation site**
		**ST**	**CC**		
1 MRSA-527	t037	ST 239	CC8	IIIA	Pus
2 MRSA-524	t037	ST 239	CC8	IIIA	Pus
3 MRSA-139	t138	ST1283	CC8	IIIA	Blood
4 MSSA-12E	t701	ST152	CC8	–	Haematoma
5 MSSA-22d	t548	ST5	CC5	–	Urine
6 MSSA-10E	t084	ST15	CC15	–	CSF

The isolates are well known for their ability to form stable biofilms (Salman et al., 2012). The isolates were received in the form of stock culture from the Medical Microbiology Laboratory/Faculty of Medicine and Health Sciences /UPM, which was previously garnered from Kuala Lumpur General Hospital (HKL), Malaysia. The sources of the isolates were from different infection sites. For the first experiment, approximately 5 × 10^5^ CFU/ml of log-phase' cells from the six *S. aureus* isolates were inoculated in 250 ml sterile glass bottles containing 100 ml tryptic soy broth supplemented with 1% glucose (TSBG; Merck, Darmstadt, Germany Baker, UK) and incubated at 37°C for 24 h. In the second experiment, the two MRSA isolates were cultured in a tryptic soy broth supplemented with 1% glucose and were grown aerobically in 6-well tissue culture polystyrene plates (Roskilde, Denmark) and incubated statically at 37°C for 12, 24 and 48 h using the method previously described by Stepanović et al. (2007).

### Bacterial secreted protein preparation

The cultured cells were centrifuged at 6000 × g for 15 min at 4°C in a refrigerated centrifuge after incubation. The supernatant was removed carefully and mixed with 10% tri-chloro acetic acid (TCA), and left overnight at 4°C to precipitate. After the overnight incubation, the precipitate was centrifuged at 10,000 × g at 4°C for 30 min. The supernatant was carefully decanted, and the protein pellets were washed several times by an ice-cold absolute acetone (Engelmann and Hecker, 2009). The resultant pellets were then air-dried for 3 min and solubilized with a rehydration buffer (Bio-Rad Laboratories, Ltd.). Since the isoelectric focusing was not successful, the protein preparations were cleaned with 2D Clean-up Kit (Bio-Rad Laboratories, Ltd.) to eliminate detergents, salts, lipids, phenolics, and nucleic acids. The proteins were solubilized again with Bio-Rad rehydration solution and RC DC Protein Assay Kit (Bio-Rad Laboratories, Ltd.) was used to quantify the concentration of proteins according to the manufacturer's instruction. Bovine serum albumin (BSA) was utilized as the protein standard. The solubilized proteins were utilized directly or stored at −80°C until further use.

### Two-dimension gel electrophoresis (2-DGE)

For the separation of extracellular proteins in the first dimension, 25 μg of solubilized exoproteins were passively rehydrated in 125 μl rehydration buffer containing 1% DTT (Bio-Red) for 14 h on a 7 cm IPG strip 4–7 (GE Healthcare Biosciences). Each strip was overlaid with 2 mL of mineral oil to prevent evaporation and urea crystallization. The IPG strips were then placed in an isoelectric focusing instrument (PROTEAN IEF cell). The IEF program was performed with the appropriate three-step protocol as shown in the Table S1. Each strip was then equilibrated in 2 ml SDS-equilibration buffer I (50 mMTris-HCl, pH 8.8, 6 M urea, 30% glycerol, 2% SDS, and 0.002% bromophenol blue) containing 50 mg dithiothreitol (DTT) and buffer II containing 250 mg iodoacetamide (IAA) for 15 min at room temperature. These incubation procedures were performed to reintroduce SDS and provide permanent reduction. Second-dimension SDS-PAGE was performed with 1 mm thick, 12% polyacrylamide gel according to the procedures described by Ziebandt et al. (2010) (Table S2). The IPG strips were then overlaid with agarose sealing solution (1% (w/v), 0.002% (w/v) bromophenol blue in tris-glycine SDS electrophoresis buffer) (Bio-Rad) and subjected to electrophoresis with 1x tris/glycine/SDS running buffer (Bio-Rad) at 200 V until the dye front reached the bottom of the resolving gel. The gels were stained with a silver stain plus kit (Bio-Rad Laboratories, Ltd.) according to the manufacturer's instructions and scanned with a Bio-Rad GS-800 scanner.

### Protein analysis by PDQuest software

The scans from the three independent experiments (25 μg) were compared to determine the differences in protein production between genotypically different isolates and biofilm cells at different time points. The PDQuest advanced 8.0.1 2D gel analysis software with the total density in gel image normalization method associated with parts per million (PPM) was utilized as a scaling factor (Bio-Rad). Data were normalized through the local regression model as recommended by PDQuest. Student's *t*-test (95% confidence interval) was employed in the statistical analysis to determine any significant differences in spot intensity (*p* < 0.05). For the experiment on biofilm development at 12, 24, and 48 h incubation, the protein changes in the 2D gels were considered when protein intensity was highly expressed. Overexpressed proteins occurring after 48 h were excised and kept at −80°C for further analysis.

### In-gel digestion and protein identification through liquid chromatography-mass spectrometry (LC-MS)

The procedures in this section were conducted by Proteomics International Pty. Ltd. (Broadway, Nedlands, Western Australia 6009). The gel pieces were subjected to in-gel digestion with trypsin after the protein spots were excised from a single representative of MRSA-527 that was grown at 48 h of biofilm formation. The peptides were extracted according to standard techniques (Bringans et al., 2008). The peptides were analyzed through LC MS/MS with Ultimate 3000 Nano HPLC system (Dionex) coupled with a 4000 Q TRAP mass spectrometer (Applied Biosystems). The tryptic peptides were loaded onto a 3 μm C18 PepMap100 column (LC Packings) and separated with a linear gradient of water/acetonitrile/0.1% formic acid (v/v). The Spectra were analyzed to identify proteins of interest using Mascot sequence matching software [Matrix Science] using the Ludwig NR database. For database searching the following parameters were used: Database, Ludwig NR; taxonomy, bacteria; enzyme: trypsin; mass tolerance: ±1.2 Da; MS/MS tolerance, ±0.05Da; mass value, monoisotopic; protein mass, unrestricted; and fragment mass tolerance, ±0.6 Da. One missed cleavage and variable modifications of methionine oxidation were allowed in the analysis. Protein identification was performed based on a statistically significant MOWSE score (*p* < 0.05).

## Results

### Extracellular protein analysis

**Figure 1:**
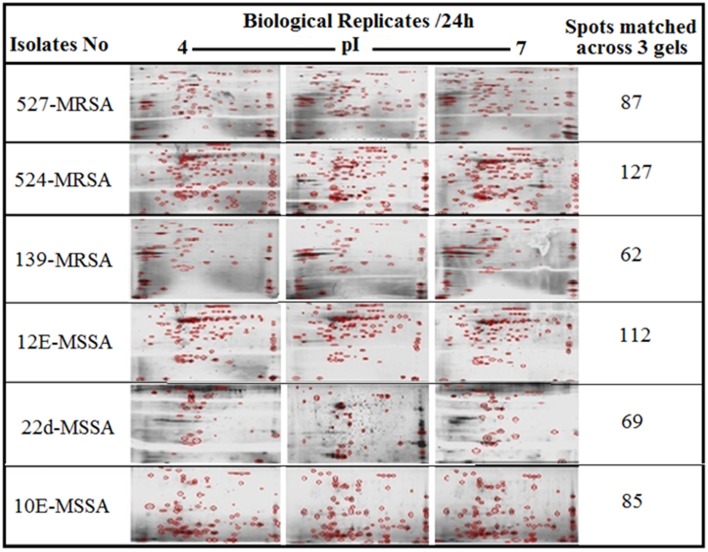
**2DE gel protein patterns of six clinical isolates of *S. aureus***. A total of 25 μg protein extract of each isolate was separated on 2D gels, using IPG strips (pI 4–7) for the first dimension. Protein spots were stained with silver stain and scanned using Densitometer GS-800 Mode Imager. PDQuest software was utilized to analyze the data in which 2DE images from the internal pooled standard from all six different isolates were employed as a reference for comparative analyses.

**Figure 2:**
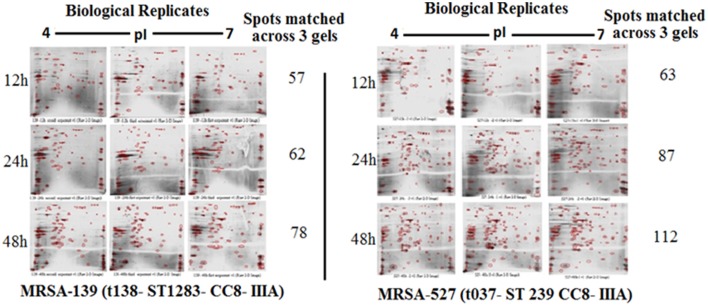
**Comparing 2DE gel protein patterns of *S. aureus* isolate number 527 and 139 under different growth time points**. A total of 25 μg protein extract of each isolate was separated on 2D gels, using IPG strips (pI 4–7) for the first dimension. Protein spots were stained with silver stain and scanned using Densitometer GS-800 Mode Imager.

**Figure 3:**
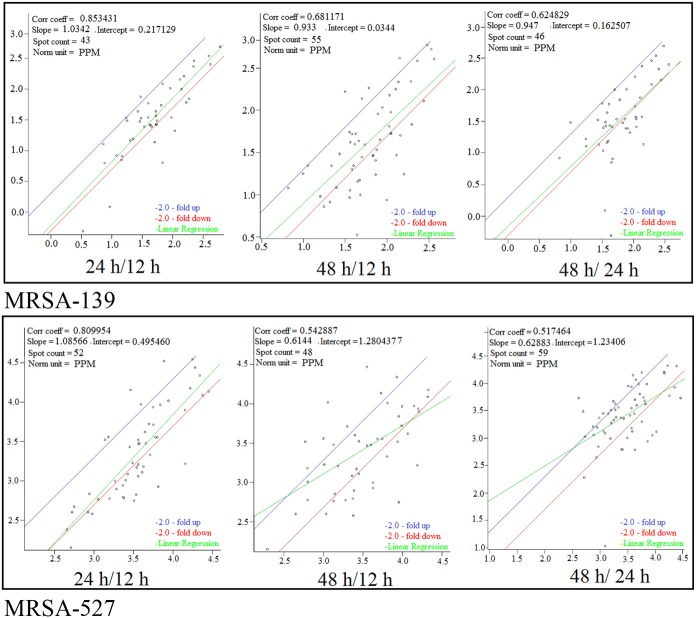
**Scatter plot of logarithmic spot quantities on three gels from different growth time points of *S. aureus* clinical isolate 527 and 139 under 12, 24, and 48 h**. Spots were normalized based on total spot quantity.

Figure 1 provides a visual of the 2DE protein profiles with a high degree of exoproteome heterogeneity among the isolates belonging to different *spa* types in the first experiment. Surprisingly, isolates with similar MLST and SCCmec types secreted proteins displayed remarkable differences either on the positional shifts (location) or number or intensity of the protein spots within the gel map. For example in descending order: isolate number 524, 12-E, 527, 10-E, 22-d, and 139 secreted 127, 112, 87, 85, 69, and 62 proteins, respectively. The overall mean coefficient of variation (C.V) was 84.13. The most obvious similarity was observed among isolates 12-E, 22-d, 524, and 527 as they shared 76, 62, 100, and 39 proteins, respectively. When the 2DE images obtained from two MRSA strains (139 and 527) at 12, 24, and 48 h incubation were imported into the PDQuest software, the number of expressed secreted proteins and spot volume were again found to be different between the isolates and even between all time-points (Figure 2). Each spot on a particular gel has to be mapped to the corresponding spot on the other gels in a process called spot matching in order to compare the spot intensities (Figure 3). Since the size and intensity of the spots differed from gel to gel; thus, a spot remodeling step was applied. This is to allow the spot boundaries to fit the gray level distributions of the original gel images and also to determine the spots that may differ significantly in terms of high levels of expression (Table 2).

**Table 2:** **Comparing high expression levels of spots from *S. aureus* clinical isolate 139 and 527, under different point time growth using PDQuest analyses software**.

**Isolates**	**Analysis set name**	**Significantly high expression spots**
MRSA-527(t037- ST 239 CC8- IIIA)	24/12 h	19
	48/12 h	21
	48/24 h	5
MRSA-139(t138- ST1283- CC8- IIIA)	24/12 h	22
	48/12 h	28
	48/24 h	8

### Proteins identified through mass spectrometry

**Figure 4:**
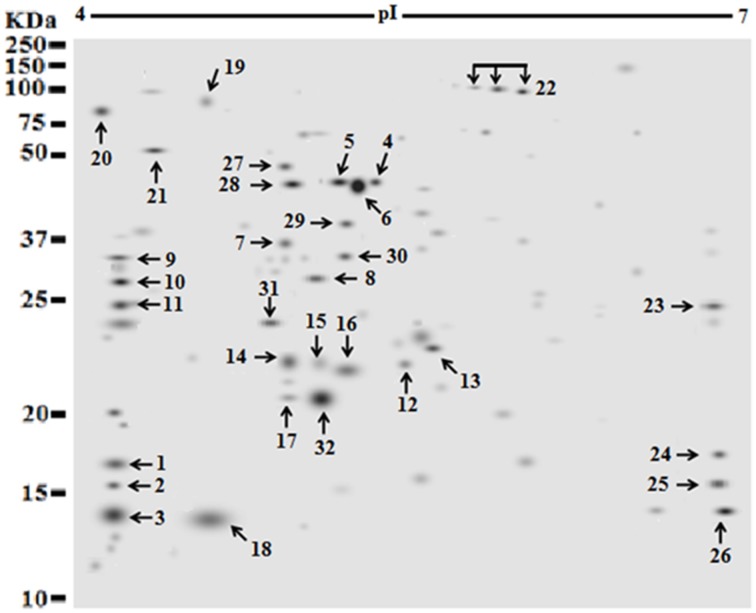
**Identification of strong expressed extracellular protein (spot) occurred after 48 h growth time points of *S. aureus* clinical isolate (527)**. The secretome of a pooled sample at 48 was separated by two dimensional electrophoresis, using IPG strip of (pI 4–7). Protein samples of 25 μg of a pooled sample from clone 527 were resolved via IEF and 7 cm format SDS-PAGE at 12% polyacrylamide concentration. Proteins were stained with silver protein gel stain and scanned by Densitometer GS-800 Mode Imager. A total of 32 selected proteins with strong expressed were manually and individually excised from the respective silver dye-stained gels and identified using LC-MS/MS after tryptic digestion.

**Figure 5:**
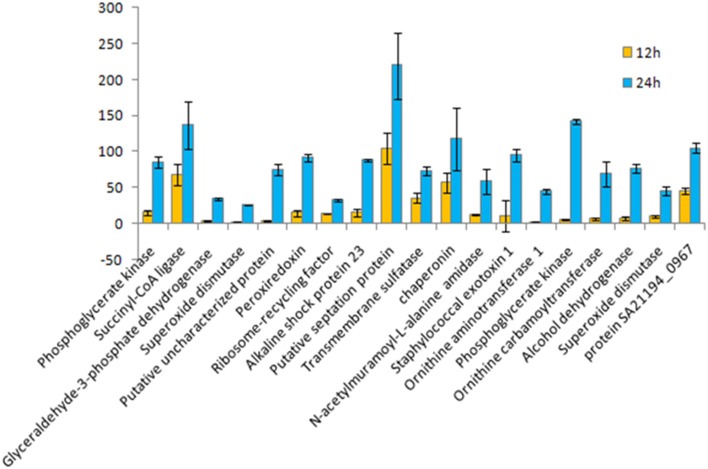
**Fold change for 12 vs. 24 h growth time points of *S. aureus* clinical isolate (527)**. A chart displaying each extracellular protein (spot) type measures the differences in protein quantity production under different growth time points of *S. aureus* biofilmdeveloped, using a PDQuest advanced Software analysis for all spots in 2DE gel. Data represent the mean of triplicate determinations of the increase in total spot quantity. An OD value of totalspot quantity in gel image normalization method associated with parts per million (PPM) was utilized as a scaling factor (Bio-Rad). Data were normalized through the local regression model as recommended by PDQuest Software. The significant difference in spot intensity was more than two-fold changes (*p* < 0.05) between 24 and 12 h.

**Figure 6:**
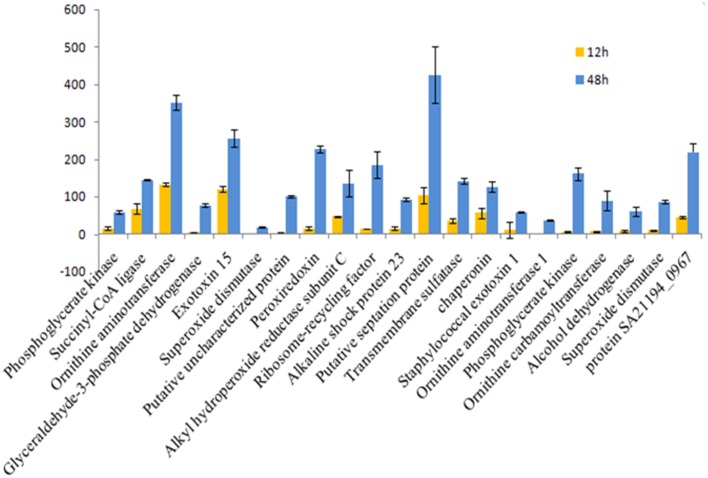
**Fold change for 12 vs. 48 hgrowth time points of *S. aureus* clinical isolate (527)**. The significant difference in extracellular protein (spot)intensity was more than two-fold changes (*p* < 0.05) between 12 and 48 h.

**Figure 7:**
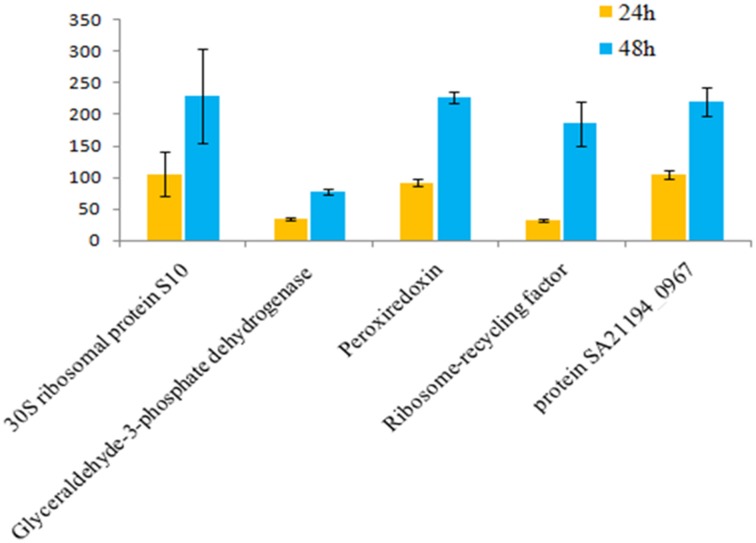
**Fold change for 24 vs. 48 hgrowth time points of *S. aureus* clinical isolate (527)**. The significant difference in extracellular protein (spot)intensity was more than two-fold changes (*p* < 0.05) between 24 and 48 h.

Of 112 differentially expressed proteins from MRSA- 527 at 48 h, 32 strongly expressed spots were selected for the identification through Mass Spectrometry using LC/MS as summarized in the Table 3 and Figure 4. In planktonic phase at 12 h incubation, there was an initial decreased in the expression of extracellular proteins expression in terms of the number spots and its intensities, and was followed by an increase of these features at 24 and 48 h of biofilm development. The highly expressed protein spots are shown in Table S3 and Figure S1. Of these, the simultaneous increase in the expression levels of protein such as alkaline shock protein 23, phosphoglycerate kinase, ligase, aminotransferase, phosphate dehydrogenase, exotoxin 15, dismutase, sulfatase, enolase, alanine amidase, aminotransferase1, phosphoglycerate kinase, and others we found in this biofilm producer, and the maximum peak of expression occurred at 48 h compared to 24 and 12 h growth phases (*p* < 0.05) as summarized in Table 2, Figures 5–7 and Figure S2. The results obtained from the LC-MS/MS were generated from mass spectrometry with several protein scores made for each protein. The peptide summary option was utilized to view the results (results can be viewed at https://sysbio-mascot.wehi.edu.au/mascot). Protein scores are the sum of a series of peptide scores which could determine the rank of each protein hit. For scores higher than 57 are considered significant at *p* < 0.05, where *p* is the probability that the observed match is a random event. The *p*-value also provides the identification number (ID) of the identified protein and the peptides that match the identified protein. The differences in protein expression with more than two-fold changes were determined for different growth time points of biofilm development.

**Table 3:** **A total of 32 strongly expression spots were identified by LC-MS/MS from *S. aureus* isolate number 527 under biofilms developed growth of 48 h**.

**Spot no**.	**Identified proteins**	**Accession no**.	**Mascot score**	**Mass Mw**	**No. of matched unique peptides**
1	50S ribosomal protein L17	H0DFF7	84	13,765	2
2	30S ribosomal protein S9	D2GJI4	125	13,485	2
3	30S ribosomal protein S10	F0P6L8	84	11,553	2
4	Phosphoglycerate kinase	D0K349	219	42,603	6
5	Succinyl-CoA ligase	I0TS27	76	41,668	2
6	Ornithine aminotransferase	F0D5L4	68	37,755	2
7	Glyceraldehyde-3-phosphate dehydrogenase	B0FZE8	77	36,268	2
8	Putative uncharacterized protein	H4GCR7	105	35,764	2
9	Exotoxin 15	H4CJ65	105	25,362	2
10	50S ribosomal protein L25	I0C1W8	108	24,320	3
11	Superoxide dismutase	I0JDL1	44	22,709	1
12	Superoxide dismutase	D2GRV5	159	22,755	3
13	Putative uncharacterized protein	H1SYF7	62	21,902	2
14	Peroxiredoxin	I0TYP8	68	21,024	1
15	Alkyl hydroperoxide reductase subunit C	Q6GJR7	161	20,963	3
16	Ribosome-recycling factor	E5RBF6	130	20,328	1
17	Alkaline shock protein 23	P0A0P6	141	19,180	4
18	Putative septation protein	F0D890	79	10,854	2
19	Transmembrane sulfatase	F0DG99	92	74,385	2
20	Chaperonin	D0K6R6	104	57,567	1
21	Enolase	D2N5I4	284	47,087	3
22	N-acetylmuramoyl-L-alanine amidase	H1TS15	283	69,208	6
23	IgG-binding protein SBI	E0P4N0	56	50,013	1
24	Epidermal cell differentiation inhibitor	P24121	53	27,663	1
25	Exotoxin 15	H4A246	153	26,305	3
26	Staphylococcal exotoxin 1	Q2YVR4	101	25,998	1
27	Ornithine aminotransferase 1	F0D9Y3	142	44,240	2
28	Phosphoglycerate kinase	H3YI93	69	42,576	3
29	Ornithine carbamoyltransferase	F0DEE9	79	37,527	2
30	Alcohol dehydrogenase	H1SYV0	61	35,926	2
31	Superoxide dismutase	I0JDL1	93	22,709	2
32	protein SA211940967	H0C9Z5	66	19,301	2

*Sequences similarity of identified proteins in the present study are available as an Ludwig NR and NCBI BLAST database. Individual ions scores >57 indicate identity or extensive homology (p < 0.05)*.

## Discussion

The influence of the clone type and the time points of biofilm growth on the extracellular protein profiles in *S. aureus* was examined by 2DE and LC-MS/MS. The progenesis PDQuest software was utilized to analyze the data in which 2DE images from the internal pooled standard from all six different isolates were employed as a reference for comparative analyses. All six *S. aureus* isolates showed significant variability in the total concentration and number of secreted proteins and they had very different 2DE gel maps, for example, isolates 10-E, 12-E, and 22-d belonging to different *spa*, MLST, and CC had different 2DE profiles, Whereas, isolates 527 and 524 belonging to the same MLST and CC also had different 2DE profiles in terms of the number of spots and its intensity produced (Figure 1), suggesting that the gene control systems may not be similar among the types of *S. aureus* clone, which could strongly disturb protein secretion and/or expression of abundant extracellular constituents. This is consistent with a previous study showing that different clinical isolates of *S. aureus* within the same CC have extremely different exoproteome profiles (Hecker et al., 2010). In the biofilm experiments, the rate of extracellular protein production was found to increase in term of spot intensity and the number of spots at 48 and 24 h compared to the exponential phase growth at 12 h incubation (Figure 2). Although this change was greater at 48 and 24 h, the maximum value of the other spots remained similar to that of spots achieved at 12 h. This may explain the increase in the rate of extracellular proteins production during biofilm growth and suggests that it can be utilized for regulation and as potential markers for the identification of biofilms. Therefore, we concur with the previous finding which suggested that a change in the growth status and culture medium causes a change in the relative rate of extracellular protein formation (Yang et al., 2014). This observation is clearly relevant in biofilm development; thus, further research should be conducted to identify the genes which are responsible for encoding these extracellular proteins. The main difficulty encountered through Mass Spectrometry in our study is that it was performed to analyse the type of secreted proteins. However, some of these proteins were found to be highly expressed at different time points of the biofilm growth which are not described as exoproteins, and have cytosolic localization (Table S3, Figure 4 and Figure S1). Hence, the presence of these proteins in culture supernatants may be due to death and/or lysis of biofilm cells during maturation. However, the alkaline shock protein 23 ‘asp23’ was found to be increased with a higher expression level in older biofilms (*p* < 0.05) (Figures 5, 6, Figure S2). To our knowledge, this finding is very interesting and corroborate with Goerke et al. (2005) in which alkaline shock protein 23 activity would peak upon entry into the stationary growth phase when *S. aureus* is grown under stressful condition and it has been confirmed that the upregulation of σ^B^ took place in the older biofilms (Goerke et al., 2005). The alternative sigma factor, σ^B^, and the accessory global regulator locus, *agr*, are the two important virulence regulatory genes, which regulate the expression of several exoproteins and surface proteins in response to changing environmental conditions. In that way, bacteria can adapt and survive in a biofilm (Knobloch et al., 2004). Our study identified the exoproteins that were strongly expressed at 48 h incubation in only one isolate, which may not, however, be the similar exoproteins produced in other isolates types. This would make sense because six different isolates in the first experiment did not show homogeneous expression of their exoproteins. Thus, we can partially conclude that there are differences in exoproteins profiles, and their protein expression at various time points of biofilm growth among the different isolates. However, this could be used as different surrogate markers for biofilm identification within the various isolates type. Hence, this type of extracellular protein production can only be applied to the MRSA isolates in this present study. It may not be suitable for other *S. aureus* isolates, even for those that exhibit similar characteristics.

Our findings could provide new insight into the genetic background of closely related *S. aureus* isolates. A slight difference in their genetic make-ups would lead to different protein profiles. It is known that bacteria could not have a complete identical genetic background as interspecies or intraspecies transmission would continuously occur even within the same clonal lineage (Liew and Neela, 2012). In addition to the variability of the 2DE protein patterns of the different clinical isolates, the results also present differences in the quantities of individual proteins. Variations were observed in terms of spot intensity among the *S. aureus* isolates despite these isolates having the same MLST and CC type. Thus, variation in protein spot intensities may be related to the differential activities of the staphylococcal gene regulators, for instance in the case of virulence factors, the activity of staphylococcal *agr* could influence those factors (Novick, 2003). The observed variations can also be attributed either to post-translational modification (PTM) or to modification occurring during the preparation of the protein samples. All these results reflect the extraordinary capacity of *S. aureus* clones to adapt and produce biofilms in various environments.

In conclusion, the present study shows that a considerable proteomic difference exists among similar and various types of *S. aureus*. A significant variation in spot size intensities was observed in the production of extracellular proteins. This variation could possibly have contributed to the degree of virulence even within the same clonal genotype and could enhanced individual heterogeneity in the infection potential. This diversity suggests that the overexpression of extracellular protein production can only be applied as a diagnostic marker for a single clone of MRSA. It cannot be applied to other clones with or without similar characteristics. Thus, the development of a rapid and precise identification profile for each clone type in human infections is important on the outcome of patients with invasive infections, in order to prescribe the correct therapeutic or reduce empirical treatment. Further immunological studies, such as studies that employ two-dimensional immunoblotting, should be conducted to screen the sera of patients infected with various *S. aureus* clones in future.

## Conflict of interest statement

The authors declare that the research was conducted in the absence of any commercial or financial relationships that could be construed as a potential conflict of interest.

## Abbreviations

*spa*: sequence region of the protein A gene
MLST: multi locus sequence typing
SCCmec: staphylococcal cassette chromosome methicillin type gene
2-DGE: Two-dimensional Gel Electrophoresis
LC-MS/MS: Liquid chromatography tandem mass spectrometry.
