# Supplementary material for: Comparative proteomic analysis of extracellular proteins expressed by various clonal types of Staphylococcus aureus and during planktonic growth and biofilm development
Source: Front Microbiol. 2015 Jun 3;6:524. doi: 10.3389/fmicb.2015.00524 (PMC4454047; doi:10.3389/fmicb.2015.00524)
Supplement: Supplementary file 1 [file Table1.DOCX]

**Supplementary data**

**Table S1** Programing the protein IEF Cell.

| Step | Voltage | Time | Voltage-Hours | Ramp |
| --- | --- | --- | --- | --- |
| 1 | 300 | 30 min | - | Liner |
| 2 | 4000 | 2 hr | - | Liner |
| 3 | 4000 | - | 10000 | Rapid |
| Total | - | ~5 hr | ~1400 | - |
| Hold | 500 | 3 hr | - | Rapid |

**Table S2** Composition of SDS-PAGE (2 piece gels)

| Resolving Gel 12% | |
| --- | --- |
| 40% Acrylamide /Bis | 6 mL |
| 1.5 M Tris-HCL,Ph 8.8 | 3.75 mL |
| 10% SDS | 150 µL |
| dd H_2_O | 5.10 mL |
| TEMED | 12.5 µL |
| 10% APS | 12.5 µL |

**Table S3** Fold change in the total of extracellular protein spot intensity in cultures of *S. aureus* isolates number 527 grown at 12 h,24 h and 48 h using PDQuest advanced Software.

| **Spot no.** | **Identified proteins** | **Fold change in level of extracellular proteins** | | | | | |
| --- | --- | --- | --- | --- | --- | --- | --- |
|  |  | 12h | SD± | 24 | SD | 48h | SD± |
| 1 | 50S ribosomal protein L17 | 218.6 | 70.9 | 121.5 | 14.8 | 242 | 8.4 |
| 2 | 30S ribosomal protein S9 | 171.5 | 35.8 | 140.5 | 6.3 | 241 | 9.8 |
| 3 | 30S ribosomal protein S10 | 151 | 22.3 | 105 | 35.3 | 229.5 | 34.2 |
| 4 | Phosphoglycerate kinase | 15.5 | 3.5 | 85.5 | 7.7 | 58.5 | 4.1 |
| 5 | Succinyl-CoA ligase | 68 | 12.1 | 137 | 32.5 | 144.5 | 2.1 |
| 6 | Ornithine aminotransferase | 133 | 4.6 | 246.5 | 2.1 | 352.5 | 20.5 |
| 7 | Glyceraldehyde-3-phosphate dehydrogenase | 4.5 | 0.7 | 34.5 | 2.1 | 77.5 | 5.5 |
| 8 | Putative uncharacterized protein | 72.5 | 0.7 | 99 | 39.5 | 82.5 | 14.8 |
| 9 | Exotoxin 15 | 120 | 7.9 | 180 | 15.3 | 256.5 | 23.3 |
| 10 | 50S ribosomal protein L25 | 178.5 | 22.8 | 87 | 11.5 | 165.5 | 23.3 |
| 11 | Superoxide dismutase | 143.3 | 14.9 | 111 | 25.2 | 159 | 32.5 |
| 12 | Superoxide dismutase | 1.85 | 0.4 | 25.5 | 0.7 | 18 | 1.4 |
| 13 | Putative uncharacterized protein | 3.5 | 0.5 | 75 | 8.4 | 101.5 | 3.5 |
| 14 | Peroxiredoxin | 15 | 4.2 | 92 | 5.6 | 227 | 9.8 |
| 15 | Alkyl hydroperoxide reductase subunit C | 48 | 1.4 | 69.5 | 2.1 | 135 | 22.3 |
| 16 | Ribosome-recycling factor | 13.5 | 0.7 | 32.5 | 2.1 | 185.5 | 34.6 |
| 17 | Alkaline shock protein 23 | 15.5 | 4.9 | 88.5 | 2.1 | 93.5 | 4.9 |
| 18 | Putative septation protein | 104.5 | 21.9 | 219.5 | 35.9 | 427.3 | 35.7 |
| 19 | Transmembrane sulfatase | 35.3 | 7.0 | 73.3 | 6.6 | 141.5 | 8.4 |
| 20 | chaperonin | 56.6 | 13.7 | 118 | 23.5 | 127 | 14.1 |
| 21 | Enolase | 154.5 | 12.4 | 89.5 | 8.3 | 105.5 | 14.3 |
| 22 | N-acetylmuramoyl-L-alanine amidase | 12.5 | 0.7 | 59 | 17.0 | 19.5 | 4.9 |
| 23 | IgG-binding protein SBI | 86 | 14.1 | 91 | 9.8 | 100 | 18.0 |
| 24 | Epidermal cell differentiation inhibitor | 138.5 | 21.9 | 55.5 | 4.4 | 48 | 3.8 |
| 25 | Exotoxin 15 | 105 | 19.7 | 98.5 | 3.7 | 88 | 2.6 |
| 26 | Staphylococcal exotoxin 1 | 10.5 | 21.9 | 95.5 | 8.4 | 58.8 | 2.3 |
| 27 | Ornithine aminotransferase 1 | 2.5 | 0.4 | 45.3 | 3.2 | 36.3 | 1.5 |
| 28 | Phosphoglycerate kinase | 5.5 | 1.3 | 142 | 2.8 | 162 | 16.9 |
| 29 | Ornithine carbamoyltransferase | 7 | 1.4 | 69.5 | 17.6 | 89 | 16.8 |
| 30 | Alcohol dehydrogenase | 8 | 2.8 | 76.6 | 6.3 | 60.5 | 12.7 |
| 31 | Superoxide dismutase | 10.5 | 1.4 | 45.5 | 6.7 | 85.5 | 4.4 |
| 32 | protein SA211940967 | 45 | 4.2 | 105 | 7.0 | 220.5 | 23.3 |


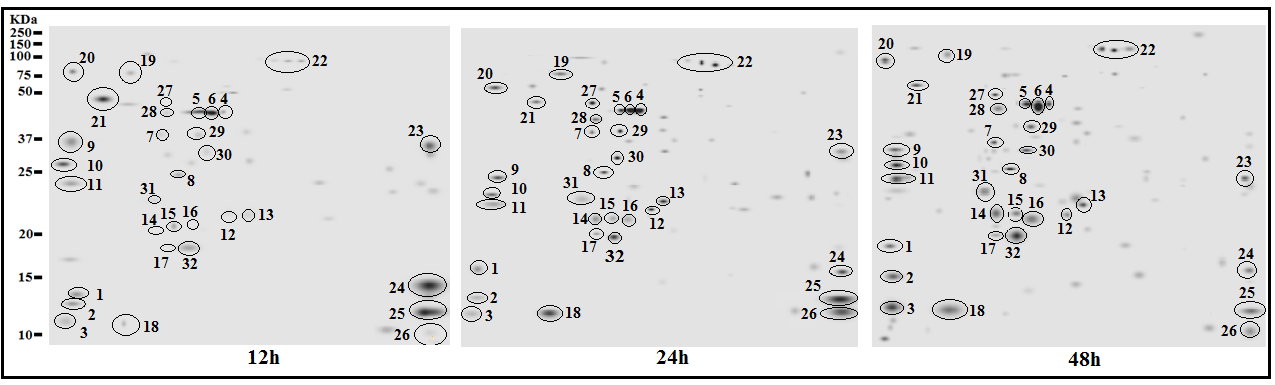


**Figure S1** Comparing the differences in the individual selected proteins spots in cultures of *S.aureus* isolates number 527 grown at12h,24h and 48h using PDQuest advanced Software analysis. The tag number indicates the spots were highly expressed compared to others of non-identified spots.


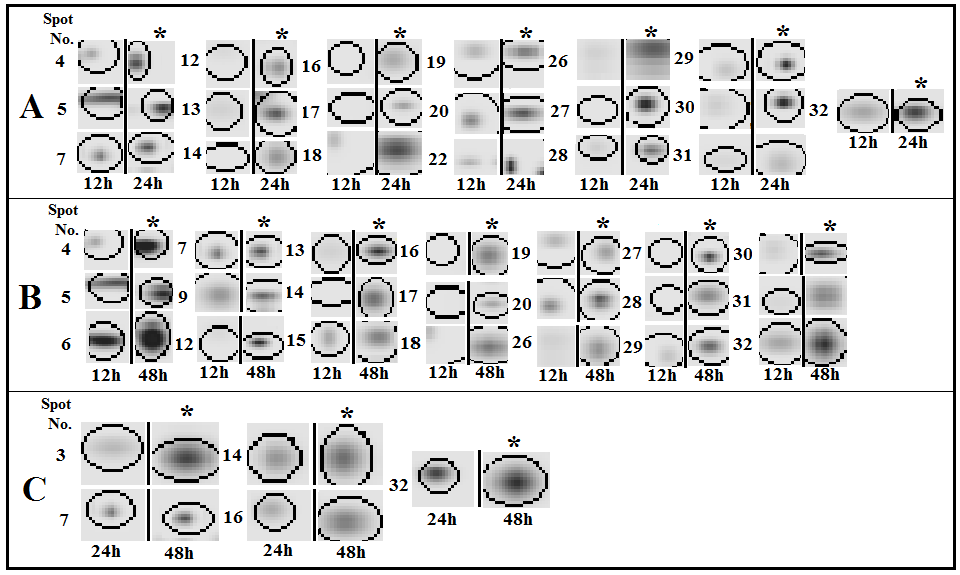


**Figure S2** Comparison of highly expressed spots pattern intensities under different point time growth of *S. aureus* biofilm isolate number 527 using PDQuest Software. A, B, C are indicated to significant difference in spot intensity more than two fold changes (p <0.05) between 12 h and 24 h; 12 h and 48 h, and 24 h and 48 h, respectively.
